# Long‐term consistency in spatial patterns of primate seed dispersal

**DOI:** 10.1002/ece3.2756

**Published:** 2017-02-04

**Authors:** Eckhard W. Heymann, Laurence Culot, Christoph Knogge, Tony Enrique Noriega Piña, Emérita R. Tirado Herrera, Matthias Klapproth, Dietmar Zinner

**Affiliations:** ^1^ Verhaltensökologie & SoziobiologieDeutsches Primatenzentrum, Leibniz‐Institut für PrimatenforschungGöttingenGermany; ^2^ Laboratório de PrimatologiaDepartamento de ZoologiaUniversidade Estadual PaulistaRio ClaroSPBrazil; ^3^ Primatology Research GroupBehavioral Biology UnitUniversity of LiègeLiègeBelgium; ^4^ Facultad de Ciencias BiológicasUniversidad Nacional de la Amazonía PeruanaIquitosPeru; ^5^ Kognitive EthologieDeutsches Primatenzentrum, Leibniz‐Institut für PrimatenforschungGöttingenGermany; ^6^ Present address: Caixa Postal 47Nazaré PaulistaSão Paulo12960-000Brazil

**Keywords:** dispersal, dispersal distances, frugivores, kernel density estimates, plant–animal interactions, tropical forest

## Abstract

Seed dispersal is a key ecological process in tropical forests, with effects on various levels ranging from plant reproductive success to the carbon storage potential of tropical rainforests. On a local and landscape scale, spatial patterns of seed dispersal create the template for the recruitment process and thus influence the population dynamics of plant species. The strength of this influence will depend on the long‐term consistency of spatial patterns of seed dispersal. We examined the long‐term consistency of spatial patterns of seed dispersal with spatially explicit data on seed dispersal by two neotropical primate species, *Leontocebus nigrifrons* and *Saguinus mystax* (Callitrichidae), collected during four independent studies between 1994 and 2013. Using distributions of dispersal probability over distances independent of plant species, cumulative dispersal distances, and kernel density estimates, we show that spatial patterns of seed dispersal are highly consistent over time. For a specific plant species, the legume *Parkia panurensis*, the convergence of cumulative distributions at a distance of 300 m, and the high probability of dispersal within 100 m from source trees coincide with the dimension of the spatial–genetic structure on the embryo/juvenile (300 m) and adult stage (100 m), respectively, of this plant species. Our results are the first demonstration of long‐term consistency of spatial patterns of seed dispersal created by tropical frugivores. Such consistency may translate into idiosyncratic patterns of regeneration.

## 1 Introduction

Seed dispersal is a key process that influences local and regional plant diversity, regeneration dynamics of plant communities, reproductive success of individual plants, spatial and genetic structure of populations at local and landscape scales, and range expansion of plant species (Levin, Muller‐Landau, Nathan, & Chave, 2003; Levine & Murrell, 2003; Nathan & Muller‐Landau, 2000; Nathan et al., 2008). In tropical forests, the majority of woody plants are adapted to zoochorous dispersal by frugivorous birds and mammals (Gentry, 1982; Peres & van Roosmalen, 2002; van Roosmalen, 1985). As large frugivores are frequently targets of hunting, and in many tropical regions their densities have been drastically reduced or they have been hunted to local extinction (Dirzo et al., 2014; Fa & Brown, 2009; Peres, 1990, 2000), seed dispersal is limited particularly for large‐seeded plants, with subsequent effects on vegetation regeneration (Effiom, Nuñez‐Iturri, Smith, Ottosson, & Olsson, 2013; Nuñez‐Iturri & Howe, 2007; Terborgh et al., 2008). This may even reverberate on the carbon stock of tropical forests (Bello et al., 2015; Osuri et al., 2016; Peres, Emilio, Schietti, Desmoulière, & Levi, 2016).

**Figure 1:**
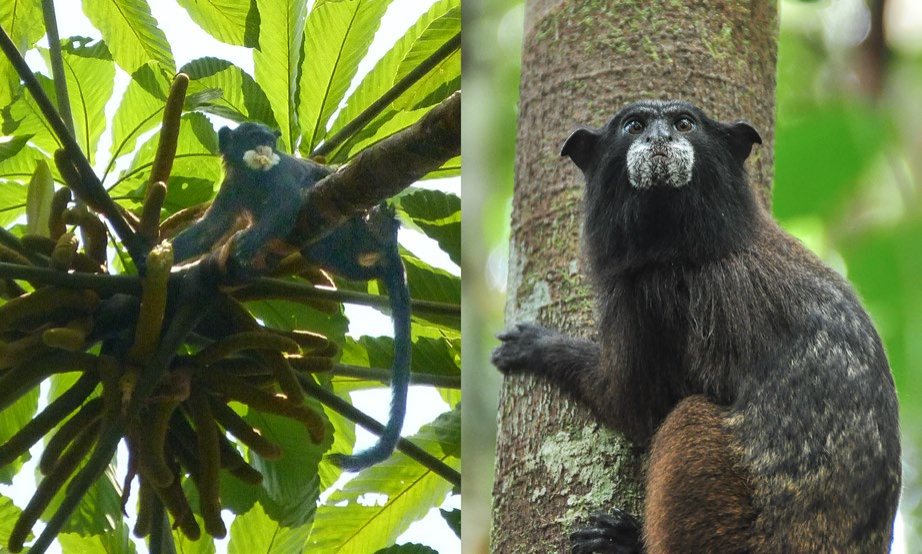
Moustached tamarins (*Saguinus mystax*, left) and saddle‐back tamarins (*Leontocebus nigrifrons*; right) are small neotropical primates that feed on a high diversity of fruits. They live in mixed‐species groups and occupy joint home ranges

Different dispersal vectors may fulfill complementary rather than redundant services, for example, with regard to quantity of dispersed seeds and to dispersal distances (McConkey & Brockelman, 2011). Thus, when vector populations decline or when vectors go extinct, their specific contribution to seed dispersal effectiveness is unlikely to be compensated by other vectors (McConkey & Drake, 2006; McConkey & O'Farrill, 2015; Schupp, Jordano, & Gómez, 2010). Effects of seed dispersal on the local and landscape level (e.g., spatial–genetic structure of plant populations) will then depend on the remaining vectors (Pérez‐Méndez, Jordano, García, & Valido, 2016). The consistency of these effects in turn depends on the spatiotemporal consistency of the seed dispersal service (Hampe, García‐Castaño, Schupp, & Jordano, 2008). Studies that examined such consistency employed trapping of seeds and sampling of recruits (e.g., Hampe et al., 2008; Houle, 1998; Nathan, Safriel, Noy‐Meir, & Schiller, 2000). In this study, we use direct observations to examine the spatiotemporal patterns of seed dispersal by two neotropical primates, the tamarins *Saguinus mystax* and *Leontocebus nigrifrons* (Callitrichidae; Figure 1). Tamarins disperse the seeds of more than 50% of the plant species they exploit for food (Culot, Muñoz Lazo, Poncin, Huynen, & Heymann, 2010; Knogge & Heymann, 2003). For their small body size (300–600 g), tamarins disperse unusually large seeds (up to 2.35 cm long and 1.35 cm wide; Knogge & Heymann, 2003). Defecations on average include 1.5 (*S. mystax*) and 1.7 seeds (*L. nigrifrons*) from 1.2 plant species (Knogge & Heymann, 2003). Here we use data from two large independent studies separated by 10 years and from two smaller independent studies to model dispersal distance distributions, calculate cumulative dispersal curves to examine the distance over which the majority of seeds are dispersed, and compare the size of dispersal kernels. In combination, this allows drawing conclusions on the long‐term consistency of seed dispersal by tamarins.

## 2 Methods

### 2.1 Study site, study groups, and field methods

We conducted field studies at the Estación Biológica Quebrada Blanco (EBQB) in northeastern Peruvian Amazonia (4°21′S 73°09′W). For details of the study area, see Heymann (1995), Knogge (1999), and Culot et al. (2010). Thirteen primate species are living at the study area and its surroundings (Table S1), but larger species are actually rare. Two major studies were conducted between March 1994 and February 1995 (1,390 observation hours on 141 days; 1,094 fecal samples for *S. mystax* and 1,376 for *L. nigrifrons*) by C.K. (Knogge, 1999) and between September 2005 and May 2008 (2,303 observation hours on 266 days over 24 months; 650 fecal samples) by L.C. (Culot, 2009). Additional data come from a study by T.E.N.P. between March and July 2013 (370 observation hours on 90 days; 212 fecal samples; Noriega Piña, unpublised thesis), and a specific study on the seed dispersal of the legume *Parkia panurensis* from May to September 2008 (635 observation hours on 67 days; Bialozyt et al., 2014; Heymann et al., 2012).

In 1994–1995, a mixed‐species group of 4–6 *L. nigrifrons* and 5–6 *S. mystax* was observed, in 2005–2008 a mixed‐species group of 3–6 *L. nigrifrons* and 4–10 *S. mystax* was observed, and in 2013 a group of 7–9 *L. nigrifrons* was observed. Groups were continuous between the 1994–1995 and 2005–2008 study periods, but individuals had completely turned over. The home range of this mixed‐species group was around 30 ha and had shifted southward by around 250 m from 1994–1995 till 2005–2008 (Figure 7). As the two tamarin species spent most of their active time in interspecific association (Heymann & Buchanan‐Smith, 2000), they can be observed simultaneously.

During observations, we collected tamarin fecal samples whenever we saw a defecating individual. Each sample was stored in a small plastic bag, tagged with a running number, time and location of defecation, and the species identity of the defecating individual. For location information, we used the trail system and mapped and tagged trees as reference points (1994–1995) and GPS recordings (2005–2013). Fecal samples were analyzed for the number and species of seeds they contained.

### 2.2 Data analyses

We considered the occurrence of one or more seeds of one plant species in a fecal sample as a single dispersal event. If seeds from two species were present in the same fecal sample, this was considered as two dispersal events. We calculated dispersal distances only when in the period between fruit consumption in an individual of a given plant species and defecation of seeds from this plant species no other individual of the same plant species was visited by the tamarins. We have recently validated this behavioral approach with genetic methods (Heymann et al., 2012). Dispersal distances were calculated as the linear distance between source plants and sites of defecation (deposition of seeds). In all comparative analyses, we included only those plant species that were represented by at least three dispersal events, to exclude plant species whose seeds are dropped by tamarins but may be swallowed accidentally.

We represented the empirical frequency distribution of dispersal distances of all plant species combined for each tamarin species and study period (1994–1995 and 2005–2008) by adjusting a nonparametric function (smooth spline curve) and its bootstrap‐estimated confidence envelope (*n* = 99 resamplings) (following Pérez‐Méndez et al., 2016). We performed the same analysis for *P. panurensis* (Fabaceae), one of the major tamarin food plants (Knogge & Heymann, 2003), only for the study periods 1994–1995, 2005–2008, and 2008; for this, we combined the seed dispersal events from the two tamarin species. We performed the analysis in R, using the script available in Jordano (2015).

We calculated cumulative dispersal distances over all dispersal events from the studies in 1994–1995, 2005–2008, and 2013. We calculated cumulative dispersal distances separately for *P. panurensis*. Data for this analysis were available from 1994–1995, 2005–2008, and 2008. For the latter period, we separately included distances obtained from observational and genetic matching of seeds to source trees (Heymann et al., 2012).

### 2.3 Kernel density estimates of dispersal events

We calculated area estimates of seed dispersal events using fixed kernel density estimates (KDE) with the rule‐based ad hoc method (Kie, 2013), hereafter SCALEDh. In contrast to the manual ad hoc choice, where REFh is reduced stepwise (0.9, 0.8, …, 0.1 of REFh), the SCALEDh approach automatically identifies the smallest bandwidth value, at which the split point is achieved (i.e., preventing contours from fragmentation). The choice of bandwidth selection rule is the most important factor influencing the probability density function, whereas the influence of kernel type is less pronounced (Wand & Jones, 1995; Worton, 1989). For both data sets, we used the standard bivariate normal function (i.e., Gaussian kernel). A cell size (i.e., resolution) of 10 m was chosen based on the small area of interest (ca. 1 km^2^), and we set the buffer extent to 0.2 to prevent the resulting contours from extending beyond the boundaries covered by the location data. We calculated respective areas enclosed by isopleths at levels from 10% to 95% in 5% steps. We chose the 95%, 50%, and 25% isopleths levels to delineate contour features in Figure 7. All calculations were carried out in R Statistics v3.3.0 (R Core Team, 2016) using the command line interface of the rhr package v1.2.906 (Signer & Balkenhol, 2015).

## 3 Results

**Figure 2:**
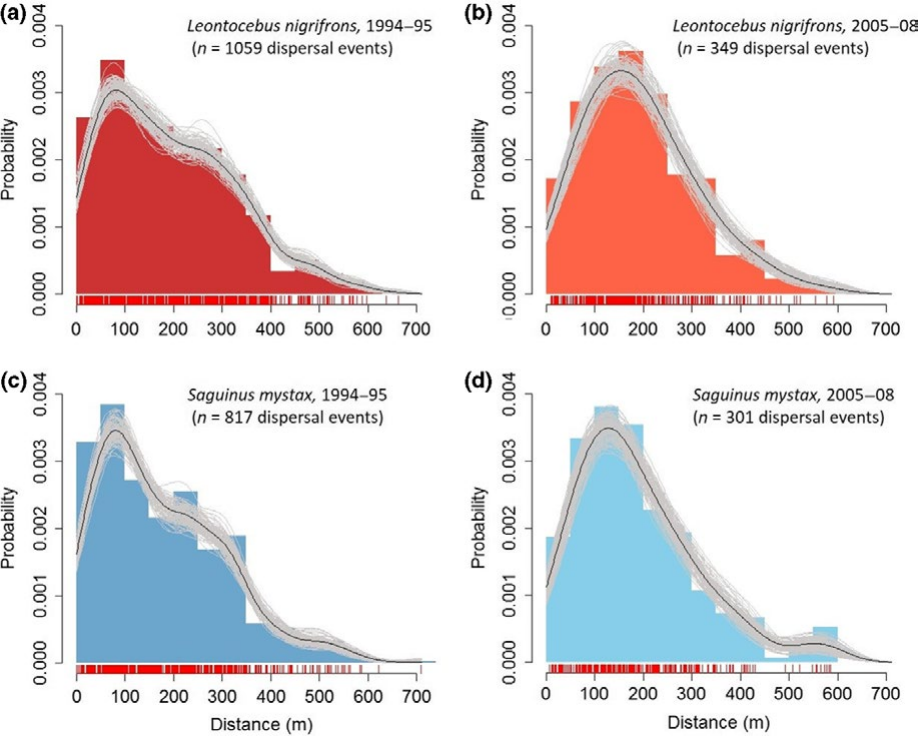
Distribution of dispersal distances (50‐m bins), all plant species combined. Red vertical bars along the *x*‐axis represent each observed dispersal event, black and gray lines a nonparametric smoothing spline fit to the empirical distance distributions together with bootstrapped estimates, respectively. (a) *Leontocebus nigrifrons*, 1994–1995; (b) *Leontocebus nigrifrons*, 2005–2008; (c) *Saguinus mystax*, 1994–1995; (d) *Saguinus mystax*, 2005–2008

**Figure 3:**
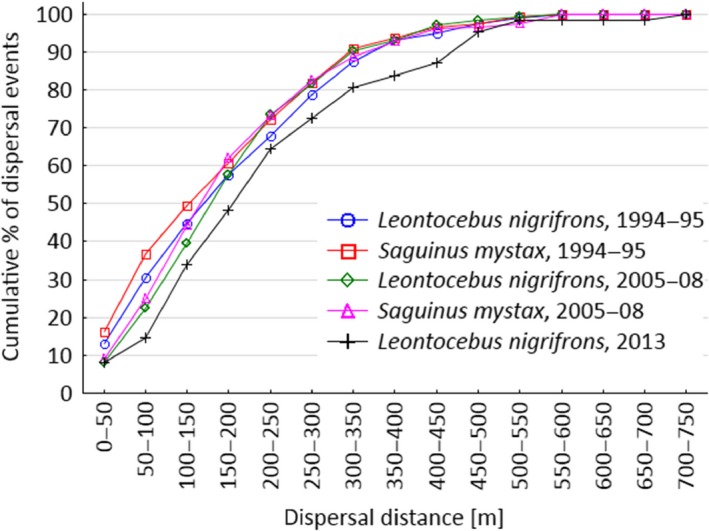
Cumulative dispersal curves for all dispersal events combined

**Figure 4:**
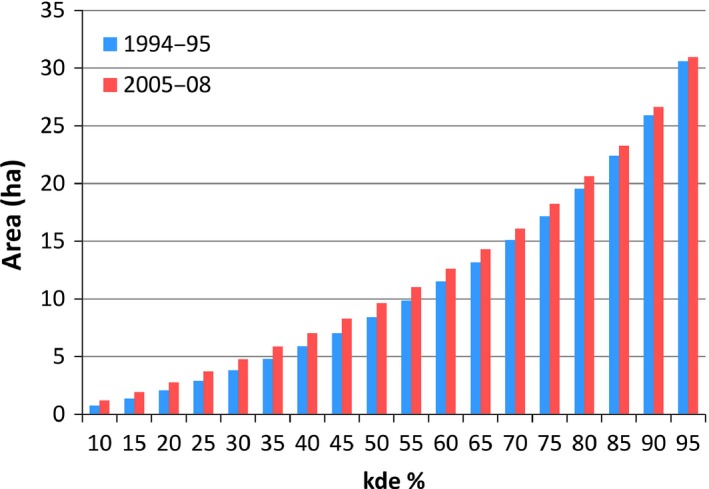
Area (ha) encompassed by kernel density estimate (KDE) isopleths for seed dispersal events in 1994–1995 (blue) and 2005–2008 (red)

Distributions of dispersal probabilities over distance, independent of plant species, are almost identical between tamarin species within a given study period and highly similar between the two major study periods (Figure 2). Cumulative dispersal distances are highly convergent between studies (Figure 3); only the 2013 study with a small sample size deviates between 300 and 400 m. The size of the area of KDE is almost indistinguishable for the same isopleths in the two different major study periods (Figure 4). Dispersal distances produced by *L. nigrifrons* ranged between 0–638 m (1994–1995; median: 173 m), 9–593 m (2005–2008; median: 183 m), and 7–730 m (2013), and in *S. mystax* between 0–709 m (1994–1995; median: 152 m) and 1–585 m (2005–2008; median: 160 m). Less than 3% of seeds are dispersed for <10 m, and only 0.6% of plants exploited by tamarins have a crown radius > 10 m.

**Figure 5:**
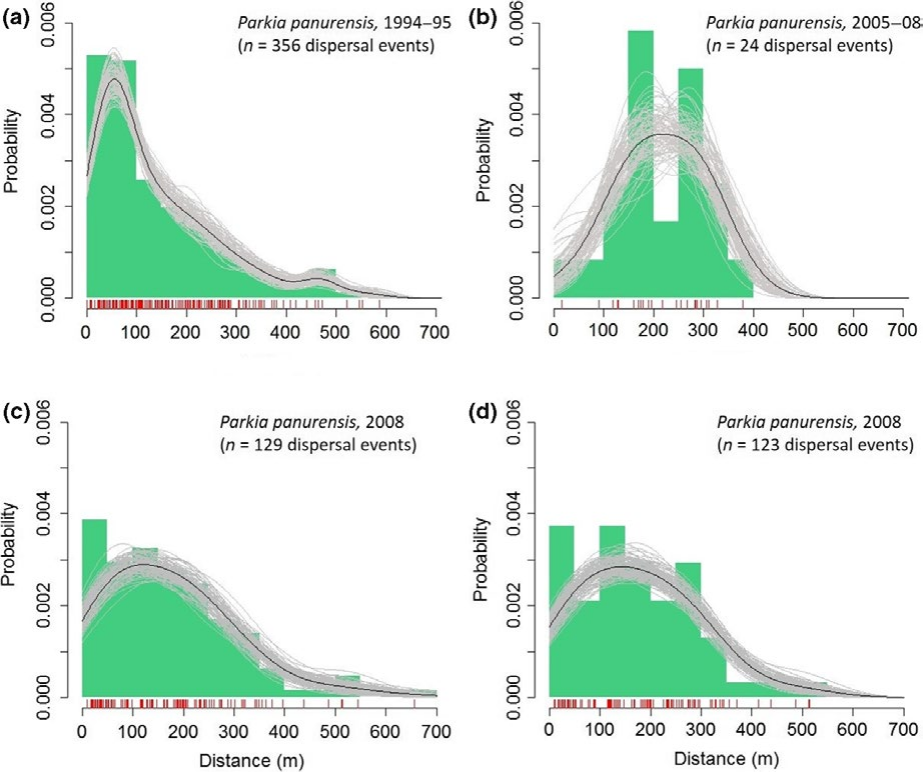
Distribution of dispersal distances (50‐m bins) for *Parkia panurensis*. Red vertical bars along the *x*‐axis represent each observed dispersal event, black and gray lines a nonparametric smoothing spline fit to the empirical distance distributions together with bootstrapped estimates, respectively. (a) 1994–1995; (b) 2005–2008; (c) 2008, based on observation; (d) 2008, based on genetic matching of seeds to source trees (Heymann et al., 2012)

**Figure 6:**
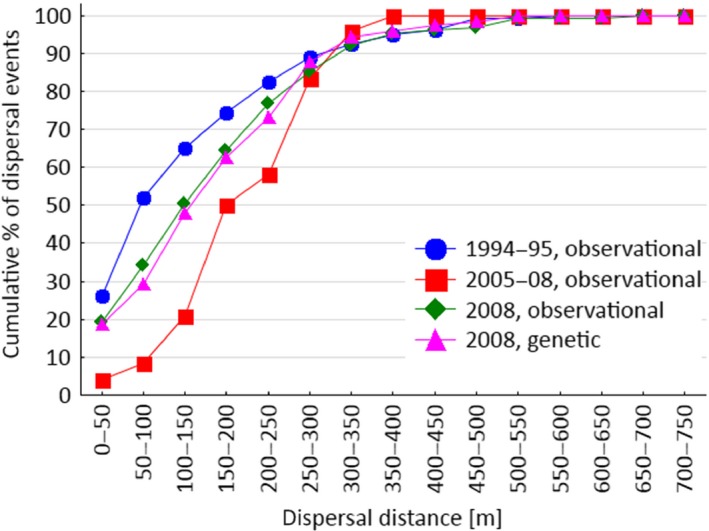
Cumulative dispersal curves for *Parkia panurensis*

For *P. panurensis,* distributions of dispersal probabilities are less consistent over different periods (Figure 5), which may be due to the small sample sizes in the 2005–2008 study and the specific study in 2008. Cumulative dispersal distances are also less consistent than those for overall seed dispersal, but strongly converge at a distance of 300 m (Figure 6). Seed dispersal distances for *P. panurensis* ranged between 0–587 m (1994–1995; median: 93 m), 91–310 m (2005–2008; median: 207 m), and 10–656 m (2008; median, observational matching: 148 m; median, genetic matching: 167 m).

**Figure 7:**
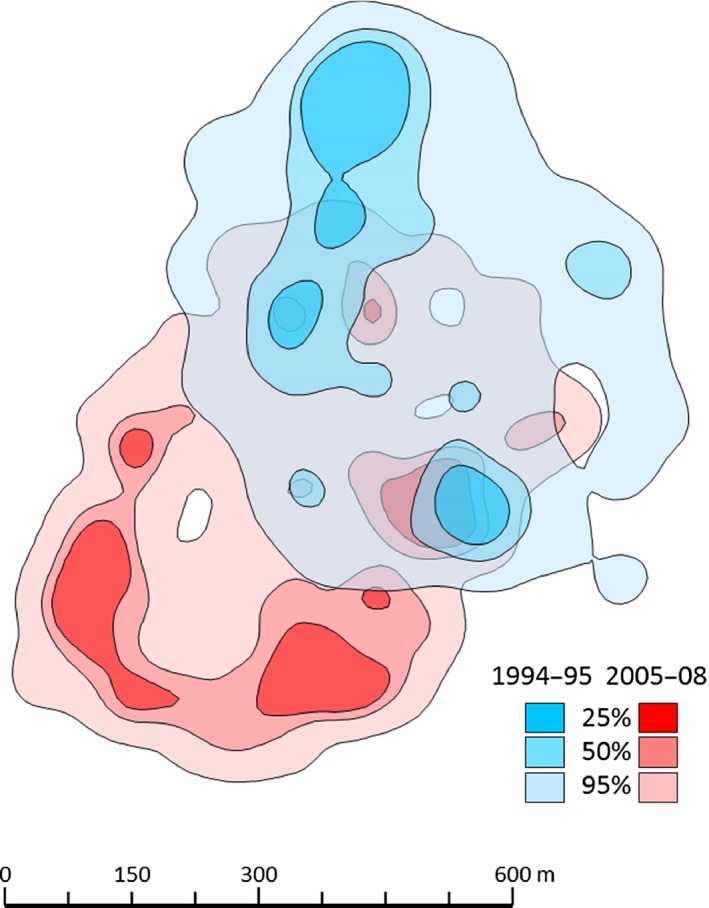
Kernel density estimates (KDE, 95%, 50%, and 25% isopleths) of seed dispersal events in 1994–1995 (blue) and 2005–2008 (red)

Different home‐range areas receive a differential seed input, but there is also overlap of kernels between 1994–1995 and 2005–2008 (Figure 7), despite the shift in home‐range area.

## 4 Discussion

Our study revealed a high degree of spatiotemporal consistency of seed dispersal by two small neotropical primates. Considering overall dispersal, independent of plant species, various measures of the spatial patterns of seed dispersal are consistent over time. For *P. panurensis,* there is variation between studies with regard to the distribution of dispersal distances, which is likely be related to discrepancies in sample size due to varied availability (phenology) of *P. panurensis* as a food resource. However, cumulative distributions are converging at a radius of 300 m. This matches the scale of the spatial–genetic population structure (SGS) on the embryo and seedling/sapling stage in the local *P. panurensis* population which is significant up to 300 m (Bialozyt et al., 2014). A significant SGS of the adult stage up to 100 m (Bialozyt et al., 2014) fits with the high probability of dispersal within this radius (Figure 6a). At EBQB, *P. panurensis* is exclusively dispersed by tamarins; it remains to be determined whether tamarin seed dispersal leaves similar genetic imprints for other plant species that tamarins disperse exclusively, like *Leonia cymosa* (Violaceae), a system currently under study. It also remains to be determined how the observed consistency affects the plant community in general. The spatial patterning of kernel density estimates suggests that there are dispersal “hot spots” (sensu Hampe et al., 2008) that persist over time. Sleeping and resting sites can be determinants of such “hot spots” (Julliot, 1997; Muñoz Lazo, Culot, Huynen, & Heymann, 2011). Whether this results in different dynamics and diversity of vegetation on a small scale is currently not known, as we do not have data on the consistency of recruitment.

While the lack of large seed dispersers obviously has strong impacts for plant species that depend on them, from vegetation diversity to the carbon storage potential of tropical forests (see section “Introduction”), it may also have impacts on plant species that loose only part of their disperser spectrum. Tamarins and woolly monkeys overlap in the plant species they exploit for fruit pulp (Peres, 1993, 1994). But while tamarins disperse seeds up to almost 700 m, the much larger woolly monkeys disperse seeds up to 1,500 m or more (Stevenson, 2000; Stevenson, Link, Onshuus, Quiroz, & Velasco, 2014). The lack of long‐distance dispersal events, even though they are generally rare (Nathan, 2006), reduces the scale of gene flow and thus impacts on the evolutionary dynamics of plant species. Whether such impacts are negative (e.g., increased risk of inbreeding) or positive (e.g., enhanced local adaptation) depends on the respective plant species' life history.

Tamarins can persist in anthropogenically disturbed and secondary forests. They have been shown to disperse seeds from primary into secondary forest (Culot et al., 2010; Oliveira & Ferrari, 2000). While they cannot compensate for the lack of seed dispersal by large primates, they thus contribute to the natural regeneration both of primary, secondary, and disturbed forests. Information on the spatiotemporal consistency should allow forecasting the long‐term effects of their seed dispersal on both individual plant species and on plant communities. However, for a more comprehensive understanding, it will be essential that similar information emerges for entire seed dispersal networks.

## CONFLICT OF INTEREST

None declared.
