# Supplementary material for: Long‐term consistency in spatial patterns of primate seed dispersal
Source: Ecol Evol. 2017 Feb 4;7(5):1435–41. doi: 10.1002/ece3.2756 (PMC5330868; doi:10.1002/ece3.2756)
Supplement: Supplementary file 1 [file ECE3-7-1435-s001.docx]

**TABLE S1**. Primate species at EBQB (ordered by increasing body mass), with information on their dietary categorization, presence in the study area and their role as seed dispersers

| **Species** | **Dietary categorization** | **Presence in area** | **Seed dispersal** |
| --- | --- | --- | --- |
| *Cebuella pygmaea* | exudativore | rare | ? |
| *Leontocebus nigrifrons* | frugivore-insectivore | very common | very frequent |
| *Saguinus mystax* | frugivore-insectivore | very common | very frequent |
| *Callicebus cupreus* | frugivore-granivor | very common | occasional |
| *Aotus nancymaae* | frugivore-insectivore | very common | frequent? |
| *Saimiri macrodon* | frugivore-insectivore | common | frequent? |
| *Pithecia monachus* | granivore | very common | rare |
| *Cebus unicolor* | frugivore-insectivore | rare | very frequent? |
| *Sapajus macrocephalus* | frugivore-insectivore | common | frequent? |
| *Cacajao calvus ucayalii* | granivore | rare | rare |
| *Alouatta seniculus* | folivore-frugivore | very rare | frequent |
| *Ateles chamek* | frugivore-folivore | very rare | very frequent |
| *Lagothrix poeppigii* | frugivore-folivore | very rare | very frequent |
